# Myocardial revascularization at extreems - Vojvodina STEMI network

**DOI:** 10.1186/1749-8090-8-S1-O185

**Published:** 2013-09-11

**Authors:** R Jung, V Ivanović, M Petrović, T Čanji, I Srdanović, S Kačar

**Affiliations:** 1 Cardiological department, Institute for Cardiovascular diseases of Vojvodina, Novi Sad, Serbia

## Background

In order to improve health care of patients with acute myocardial infarction, forming of a proper cardiological network in different parts of a particular region becomes a necessity.

At our institute, we started to treat STEMI in cath-labs in April 2002. In the beginning this care has been utilized during the day, and after the 1. 12. 2008, we started with 24-hours practice, so at this moment we are talking about 4 years’ experience.

After the one year, we concluded that we can increase the number of patients. The connection between cities in Vojvodina started in 2009, at first with Vrbas, and then, depending on the infrastructure, we gradually made a whole network between the cities and medical centers in almost whole part of the country. Nowadays we are connected with 11 medical centers in Vojvodina. The guidelines (2010) supported our proper orientation.

Using that network, in the last 4 years we have catheterised 3123 patients with STEMI. In the first 2 hours of STEMI 2573 patients were treated in our cath-lab (in 2012 – 951 patients).

We do not select patients – all the patients with STEMI have to go to a cath-lab and be treated with PCA. The youngest patient was 26, and the oldest was 93 years old.

TIMI flow III has been achieved in 91.9% of patients. 2363 of 2573 has been stented (73.6% BMS, 18.2% DES), and 210 patents (8.2%) has been only dilated.

In the case of the unsuitable coronary anatomy for PCI, we are using the advantage of a presence of cardiac surgery.

The hospital mortality in Coronary Care Unit of all STEMI patients was less than 10%.

